# Combination Treatment of OSI-906 with Aurora B Inhibitor Reduces Cell Viability via Cyclin B1 Degradation-Induced Mitotic Slippage

**DOI:** 10.3390/ijms22115706

**Published:** 2021-05-27

**Authors:** Yuki Ikeda, Ryuji Yasutake, Ryuzaburo Yuki, Youhei Saito, Yuji Nakayama

**Affiliations:** Department of Biochemistry & Molecular Biology, Kyoto Pharmaceutical University, Kyoto 607-8414, Japan; kd21001@ms.kyoto-phu.ac.jp (Y.I.); kd21008@ms.kyoto-phu.ac.jp (R.Y.); yuki2019@mb.kyoto-phu.ac.jp (R.Y.); ysaito@mb.kyoto-phu.ac.jp (Y.S.)

**Keywords:** linsitinib, OSI-906, ZM447439, IGF1R, Aurora B, M phase, RO-3306

## Abstract

Insulin-like growth factor 1 receptor (IGF1R), a receptor-type tyrosine kinase, transduces signals related to cell proliferation, survival, and differentiation. We recently reported that OSI-906, an IGF1R inhibitor, in combination with the Aurora B inhibitor ZM447439 suppresses cell proliferation. However, the mechanism underlying this suppressive effect is yet to be elucidated. In this study, we examined the effects of combination treatment with OSI-906 and ZM447439 on cell division, so as to understand how cell proliferation was suppressed. Morphological analysis showed that the combination treatment generated enlarged cells with aberrant nuclei, whereas neither OSI-906 nor ZM447439 treatment alone caused this morphological change. Flow cytometry analysis indicated that over-replicated cells were generated by the combination treatment, but not by the lone treatment with either inhibitors. Time-lapse imaging showed mitotic slippage following a severe delay in chromosome alignment and cytokinesis failure with furrow regression. Furthermore, in S-trityl-l-cysteine–treated cells, cyclin B1 was precociously degraded. These results suggest that the combination treatment caused severe defect in the chromosome alignment and spindle assembly checkpoint, which resulted in the generation of over-replicated cells. The generation of over-replicated cells with massive aneuploidy may be the cause of reduction of cell viability and cell death. This study provides new possibilities of cancer chemotherapy.

## 1. Introduction

Insulin-like growth factor 1 (IGF1) receptor (IGF1R), a receptor-type tyrosine kinase [1], is generated by cleavage of its precursors. IGF1R is composed of two α and two β subunits. When bound with IGF1, the kinase activity of IGF1R is activated to trigger the signaling pathways for cell proliferation, differentiation, and survival. The auto-phosphorylated tyrosine residues serve as a docking site for insulin receptor substrate 1 and Shc and trigger downstream signaling, including Raf/mitogen-activated protein kinase (MEK)/extracellular signal-regulated kinase pathways and phosphatidylinositol 3-kinase (PI3K)/AKT. It has been reported that IGF1R expression levels correlate with progression of cancer and metastatic phenotypes [2,3,4,5,6,7,8]. This is supported by the fact that increases in plasma IGF1 levels are associated with cancer risk [9,10,11,12]. Recently, we reported an abnormal cell division upon inhibition of receptor-type tyrosine kinases, including EphA2 [13], ALK [14], VEGF receptor [15], and IGF1R [16].

Cell division is among the most complicated cellular processes, in which replicated chromosomes are divided into two daughter cells. Ser/Thr kinases, such as cyclin dependent kinase 1 (CDK1), Aurora kinases, and Polo-like kinase, orchestrate various events to promote this process [17]. At the onset of cell division, DNA is condensed into compact chromosomes. The nuclear envelope breakdown permits microtubules to approach chromosome’s kinetochore. Powered by motor proteins and polymerization and depolymerization of microtubules, chromosomes converge at the cell equator and align at the metaphase plate. Incorrect attachment of microtubules stimulates the spindle assembly checkpoint (SAC) to prevent the onset of anaphase. Upon satisfaction of SAC, cohesin maintaining sister chromatid cohesion degrades and sister chromatids segregate to opposite poles. The spindle midzone forms between the segregating chromosomes and the proteins that regulate cytokinesis localize there. Chromosomes are decondensed at the poles and the nuclear envelope reassembles. Finally, the cytoplasm splits into two daughter cells.

Anti-mitotic agents have been commonly used for chemotherapy against a wide range of cancers. Disruption of microtubule dynamics is an effective way to interrupt progression of cell division. Taxanes and vinca alkaloids are traditional anti-mitotic agents. Taxanes are known to stabilize microtubules. Conversely, vinca alkaloids depolymerize them at higher concentrations and inhibit microtubule dynamics at lower concentrations. They have been successfully used in clinical. However, side effects and drug resistance limit the usefulness of these anti-mitotic drugs, which has led to the development of new generation of microtubule poisons as well as agents targeting mitotic kinases and microtubule motor proteins. Thus, combination therapy of anti-mitotic drugs with compounds targeting cancer-specific alterations must be useful to reduce the side effects. Recently, we reported that targeting IGF1R combined with Aurora B inhibition suppresses cell proliferation more than single treatment [16]. However, the molecular mechanisms underlying the suppressive effect of this combination on cell proliferation is yet to be elucidated.

In this study, we investigated the mechanisms underlying the suppression of the cell proliferation. Cell cycle synchronization experiments and time-lapse imaging revealed that combination treatment of OSI-906 and ZM447439 caused severe defect in the chromosome alignment and SAC function. The resulting generation of over-replicated cells with massive aneuploidy may be responsible for the effects of the combination of OSI-906 and ZM447439 on cell proliferation.

## 2. Results

### 2.1. Combination of OSI-906 and ZM447439 Causes Cell Death via Generation of Enlarged Cells with Aberrant-Shaped Nuclei

**Figure 1:**
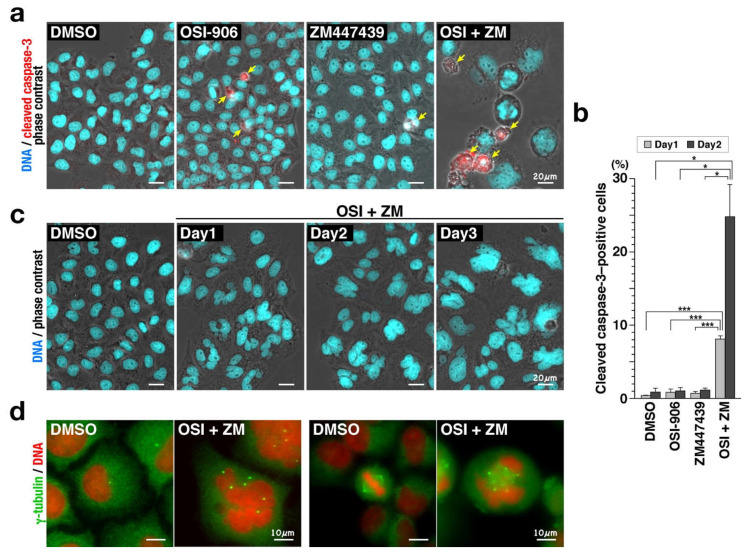
Combination of OSI-906 and ZM447439 generates enlarged cells with aberrant-shaped nuclei. HeLa S3 cells were treated with 3 µM OSI-906, 1 µM ZM447439, or their combination for 3 days. The cells were fixed with methanol for 5 min at −30 °C (**a**,**c**,**d**) or 4% formaldehyde for 20 min at room temperature (**b**). (**a**) The fixed cells (Day3) were stained for cleaved caspase-3 (red) and DNA (cyan). Representative images of cells treated with OSI-906, ZM447439, or their combination (OSI + ZM) are shown. Dimethyl sulfoxide (DMSO) was used as solvent control. Yellow arrows indicate cleaved caspase-3–positive cells. Scale bar, 20 µm. (**b**) On day 1 and day 2, the cells were fixed and stained for cleaved caspase-3 and DNA. The number of cleaved caspase-3–positive cells were counted and plotted as the mean ± SD calculated from three independent experiments (n > 201). *p*-values were calculated using Tukey’s multiple comparison test (Day1) and the Games–Howell multiple comparison test (Day2). * *p* < 0.05; *** *p* < 0.001. (**c**) The fixed cells were stained for DNA (cyan). Representative images of cells treated with the combination (OSI + ZM) for 1–3 days are shown. Scale bar, 20 µm. (**d**) The fixed cells (Day3) were stained for γ-tubulin (green) and DNA (red). Representative images of γ-tubulin in interphase (left) and M phase cells (right) treated with the combination (OSI + ZM) are shown. Scale bar, 10 µm.

A retrospective analysis of patients with cervical cancer showed that IGF1/IGF1R signaling is involved in tumor formation and clinical outcome [8]. We previously reported that although 3 µM OSI-906 suppressed 48% of cell proliferation, combination with 1 µM ZM447439, which caused almost no suppressive effect, suppressed 74% of cell proliferation of cervical cancer HeLa S3 cells [16]. However, the precise mechanism behind this effect is yet to be elucidated. Therefore, we first examined cell morphology during the combination treatment. Immunofluorescence staining after 3-day treatment revealed that the combination treatment of cells with OSI-906 and ZM447439 generated enlarged cells with aberrant-shaped nuclei (Figure 1a). The nuclei were much larger than the cells treated with either OSI-906 or ZM447439 (Figure 1a). Furthermore, the cleaved caspase-3–positive cells were observed in the cells treated by inhibitors and the number of cleaved caspase-3–positive cells were significantly increased upon the combination treatment (Figure 1a,b). Considering that the number of cells after 72 h of combination treatment was less than that of seeded cells (Figure 7B in [16]), these results suggest an involvement of cell death in suppression of cell proliferation. It is worthy to note that the cleaved caspase-3–positive cells generated by the combination treatment appear to be larger than those obtained by either OSI-906 or ZM447439 treatment (Figure 1a). The nuclear size increased with time and their morphology was complex and highly variable (Figure 1c). The number of γ-tubulin foci showed marked differences between control and the combination-treated cells. In the combination-treated cells, enlarged cells with large and aberrant-shaped nuclei exhibited an increased number of centrosomes (Figure 1d, left). In addition, multipolar spindle was found in M phase upon the combination treatment, whereas bipolar spindle was observed in solvent control cells (Figure 1d, right). Taken together, these results suggest that combination treatment may induce cell death via generation of enlarged cells with aberrant-shaped nuclei and via increase in the number of centrosomes.

### 2.2. Combination of OSI-906 and ZM447439 Generates over-Replicated Cells

**Figure 2:**
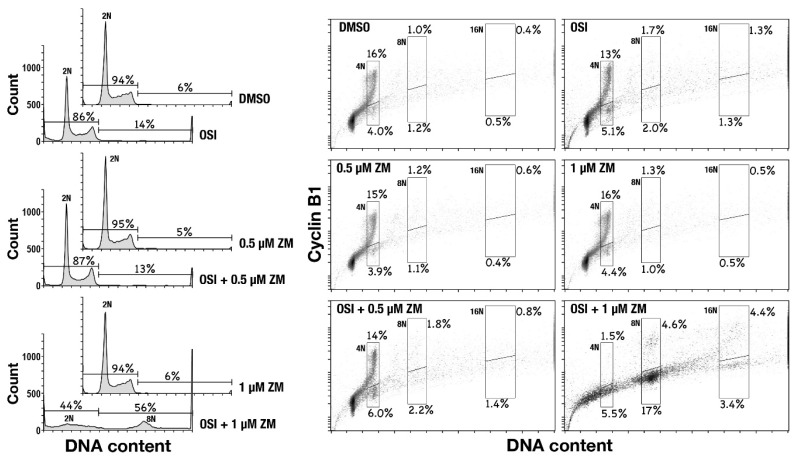
Combination of OSI-906 and ZM447439 causes over-replication. HeLa S3 cells were treated with 3 µM OSI-906, 0.5 or 1 µM ZM447439, or their combination for 48 h. The cells were detached from dishes, fixed with 70% ethanol for 1 h at –30 °C, and stained for cyclin B1 and DNA. DNA histograms are shown on the left. Peak haploid genome equivalents are indicated. Percentages of cells with more than 4N DNA content and less than that of DNA content are shown. Each plot represents 18,000 cells. The bivariate dot plots of 18,000 cells are shown on the right; DNA content is shown on the x-axis (linear scale), while cyclin B1 protein level is shown on the y-axis (log scale). Regions designated by line include cells having 4N, 8N, or 16N DNA content, while lower and upper regions include cyclin B1-negative and -positive cell populations, respectively. Percentage of cell numbers within the region to the whole cell number is shown near the region.

An increased number of centrosomes is a hallmark of DNA over-replication. Therefore, to examine whether enlarged cells contained an excess amount of DNA, cells were treated with either OSI-906 or ZM447439, or their combination for 48 h and analyzed for DNA content and cyclin B1 levels by flow cytometry. Control cells (DMSO) showed a typical pattern of DNA content. Upon combination treatment, cells exhibited over-replicated DNA more than 4N (Figure 2, left), suggesting that enlarged cells contain over-replicated DNA more than 4N. Aurora B plays a role in the SAC. Therefore, inhibition of Aurora B was expected to induce mitotic slippage, thus generating cells with over-replicated DNA. However, ZM447439 treatment, at the concentration used in this study, did not generate cells having more than 4N DNA content. 

Bivariate plot of cyclin B1 levels versus DNA content revealed an increase in the number of 4N, 8N, and 16N cells with lower cyclin B1 levels (Figure 2, right). Since cyclin B1 accumulates through G2 and degrades at the onset of anaphase, these cells with lower cyclin B1 levels would be anaphase/telophase-arrested cells or cells generated by mitotic exit without cytokinesis. Based on the result shown in Figure 1, in which the number of enlarged cells with aberrant-shaped nuclei was increased, these results suggest that 4N, 8N, and 16N cells with low cyclin B1 levels may be generated by mitotic exit without cytokinesis or with cytokinesis failure.

### 2.3. Defect in Chromosome Alignment upon Combination Treatment

**Figure 3:**
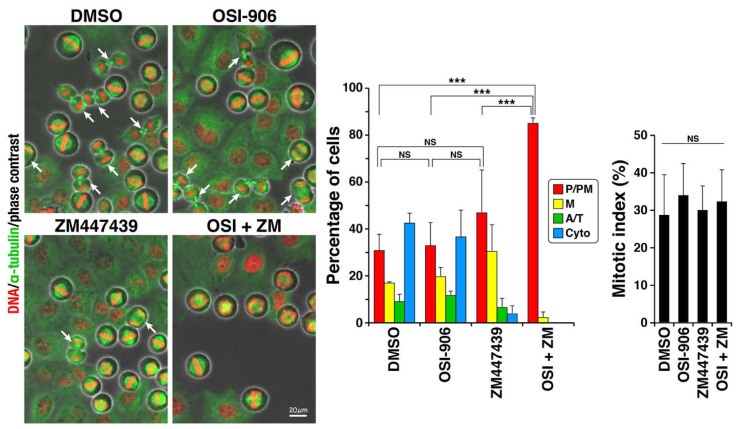
Combination of OSI-906 and ZM447439 causes severe defect in chromosome alignment. HeLa S3 cells were treated with 6 µM RO-3306 for 20 h, released by washing out this drug, and then cultured without RO-3306. After 60-min culture, the cells were fixed and stained for α-tubulin and DNA. Based on morphologies of spindle and chromosomes, mitotic cells were classified into four groups: prophase/prometaphase (P/PM, red), metaphase (M, yellow), anaphase/telophase (A/T, green), and cytokinesis (Cyto, blue). Cells were treated with 3 µM OSI-906, 1 µM ZM447439, or their combination (OSI + ZM) for 1 h at the end of the RO-3306 treatment and were continuously treated during the 60-minute release. Dimethyl sulfoxide was used as solvent control (DMSO). Representative images are shown. Arrows indicate cells after the onset of anaphase. The average value of each group and the mitotic index, a ratio of mitotic cells among total cell numbers, were calculated from three independent experiments (n > 222 in each experiment) and expressed as mean ± SD. *p*-values were calculated using Tukey’s multiple comparison test. *** *p* < 0.001. NS, not significant.

We previously reported that OSI-906 delays M phase progression [16]. ZM447439 is an inhibitor of Aurora B, a protein that is critical for M phase progression. To scrutinize how this combination treatment generated over-replicated cells, we first examined the effect of OSI-906, ZM447439, or their combination at the same concentration used in Figure 1 and Figure 2 on M phase progression (Figure 3). Cells were treated with the CDK1 inhibitor RO-3306 for 20 h to be stopped at the G2/M border and released from G2/M arrest [15,18]. In this protocol, approximately 30–40% of the cells synchronously enter the M phase, and the other cells do not enter the M phase for at least 2 h after being released from the drug treatment. Therefore, the M phase progression rates can be compared. In the present study, approximately 30% of the cells entered the M phase, with no differences among the inhibitors. Classification of M phase cells into four groups showed that 43% of control cells progressed to cytokinesis and 31% of cells did not align chromosomes. In sharp contrast, the combination of OSI-906 and ZM447439 severely affected chromosome alignment, as 97% of the cells were still in prophase/prometaphase. ZM447439 at 1 µM slightly, but not significantly, increased prophase/prometaphase cells. In contrast, OSI-906 alone at 3 µM did not delay the M phase progression at this concentration. Therefore, the effect of the combination treatment was not attributed to their additive effect. We did not expect that 1 µM ZM447439 delayed M phase progression, since ZM447439 at this concentration did not affect cell morphology (Figure 1), DNA content (Figure 2), and cell proliferation [16]. It is assumed that although 1 µM ZM447439 delays the M phase progression, cell may successfully divide into two daughter cells. These results suggest that severe defect in chromosome alignment may be among the causes of over-replication of cells upon the combination treatment, although it is not sufficient to cause over-replication.

### 2.4. Mitotic Slippage and Cytokinesis Failure

**Figure 4:**
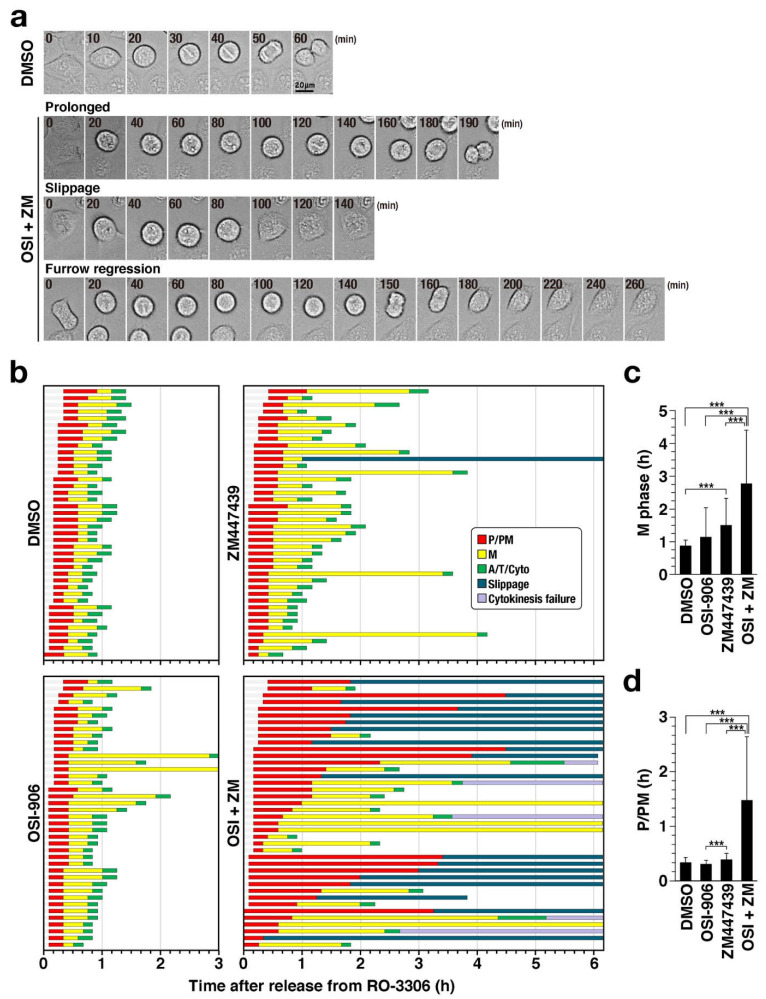
Combination of OSI-906 and ZM447439 causes mitotic slippage and cytokinesis failure. HeLa S3 cells were treated with 6 µM RO-3306 for 20 h and with either 3 µM OSI-906, 1 µM ZM447439, or their combination (OSI + ZM) for 1 h at end of the RO-3306 treatment. The cells were released by washing out the drug and then cultured continuously with inhibitors without RO-3306. Immediately after the release from RO-3306 treatment, time-lapse imaging was started. Images of bright field and Hoechst 33342 fluorescence (DNA) were captured every 5 min. (**a**) Representative images of cells with prolonged M phase, mitotic slippage, and furrow regression are shown. (**b**) The mitotic cells were classified based on their morphologies of cell shape and chromosomes into three groups: prophase/prometaphase (P/PM), metaphase (M), and anaphase/telophase/cytokinesis (A/T/Cyto). Based on the time-lapse images, the duration of each mitotic sub-phase (P/PM, M. A/T/Cyto) in individual cells are plotted in the graph (n = 40). Cells that exited mitosis without chromosome segregation and cytokinesis are shown as “Slippage.” Cells whose cleavage furrows ingressed and then regressed were shown as “cytokinesis failure.” (**c**,**d**) Mean values of the durations of M phase (**c**) and P/PM (**d**) were plotted as mean ± SD. *p*-values were calculated using the Games–Howell multiple comparison test. *** *p* < 0.001.

The combination treatment caused severe defect in chromosome alignment (Figure 3). However, this cannot completely explain how DNA was over-replicated. Therefore, cells were synchronized using RO-3306 and M phase progression was continuously observed by time-lapse imaging for 6 h (Figure 4a,b). While almost all control cells exit mitosis within 70 min, it took more than 70 min for one-fourth of OSI-906–treated cells and one-half of ZM447439-treated cells. Mean values of duration of M phase was significantly increased from 53 min (DMSO) to 167 min (OSI+ZM) (Figure 4c). As expected, the combination treatment of OSI-906 and ZM447439 caused severe delay in chromosome alignment (Figure 4b, OSI+ZM, red, Figure 4d) and prevented the onset of anaphase, even after alignment of chromosomes (yellow). Intriguingly, approximately one-half of cells underwent mitotic slippage (blue, 47.5%), where cells exited mitosis without chromosome segregation. Furthermore, a small fraction of cells failed cytokinesis (purple, 12.5%), where the cleavage furrow ingressed, but failed after chromosome segregation. These results suggest that, in addition to defects in chromosome alignment, mitotic slippage and cytokinesis failure result in the generation of over-replicated cells.

### 2.5. Precocious Degradation of Cyclin B1

**Figure 5:**
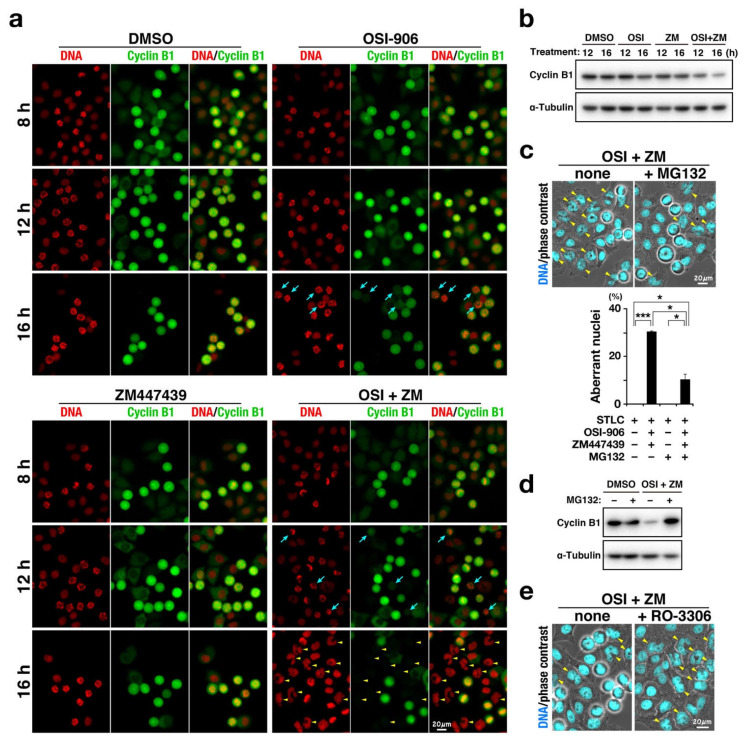
Combination of OSI-906 and ZM447439 causes mitotic slippage via precocious degradation of cyclin B1. HeLa S3 cells were treated with 7.5 µM S-trityl-L-cysteine in the presence of OSI-906, ZM447439, or their combination (OSI + ZM). Dimethyl sulfoxide was used as solvent control (DMSO). (**a**) At 8, 12, and 16 h after the start of treatment, cells were fixed and stained for cyclin B1 (green) and DNA (red). Representative images are shown. Blue arrows indicate M phase cells with lower expression levels of cyclin B1. Yellow arrowheads indicate cells with aberrant nuclear morphologies. Scale bar, 20 µm. (**b**) At 10 h after the treatment, M phase cells were collected by mitotic shake-off and further incubated in microtubes at 37 °C for 2 h and 6 h. Whole cell lysates were analyzed using Western blot with the indicated antibodies. The full blots are shown in Figure S1. (**c**) At 12 h after the treatment, 40 µM MG-132 was added to the culture. The cells were further incubated for 4 h and stained for DNA (cyan). Yellow arrowheads indicate cells with aberrant nuclear morphologies. Scale bar, 20 µm. The number of cells with aberrant-shaped nuclei was counted and plotted as the mean ± SD calculated from three independent experiments (n > 112). *p*-values were calculated using the Games–Howell multiple comparison test. * *p* < 0.05; *** *p* < 0.001. (**d**) At 10 h after the treatment, M phase cells were collected by mitotic shake-off and further incubated in microtubes at 37 °C for 6 h. During the last 4 h of incubation in microtubes, the cells were treated with 40 µM MG-132. Whole cell lysates were analyzed using Western blot with the indicated antibodies. The full blots are shown in Figure S1. (**e**) At 10 h after the treatment, 6 µM RO-3306 was added to the culture. The cells were further incubated for 2 h and stained for DNA (cyan). Yellow arrowheads indicate cells with aberrant nuclear morphologies. Scale bar, 20 µm.

We next investigated why the combination treatment caused mitotic slippage. The transition from metaphase to anaphase is regulated by the SAC. Satisfaction of SAC enables the onset of anaphase via ubiquitin/proteasome pathway–mediated degradation of a subset of proteins [19]. Cyclin B1 is one of these proteins. Therefore, cells were treated with OSI-906, ZM447439, or their combination in the presence of S-trityl-L-cysteine (STLC) to activate SAC and cyclin B1 expression level was examined (Figure 5). STLC is a reversible inhibitor of kinesin Eg5, which is responsible for formation of the bipolar spindle [20]. Inhibition of Eg5 results in formation of the monopolar spindle; the resulting incorrect microtubule attachment to the kinetochores activates SAC. The STLC treatment caused accumulation of M phase cells until 16 h. However, upon the combination treatment, cells with lower cyclin B1 expression levels were identified at 12 h and several interphase cells with aberrant-shaped nuclei were found at 16 h (Figure 5a). Western blot analysis confirmed the decrease in cyclin B1 levels upon combination treatment (Figure 5b and Figure S1). In contrast, treatment with ZM447439 at 1 µM did not affect cyclin B1 levels. Although OSI-906 treatment alone did not decrease cyclin B1 levels at 12 h, a decrease in cyclin B1 levels was observed at 16 h (Figure 5a). In addition, simultaneous treatment with MG132 prevented the generation of cells with aberrant-shaped nuclei (Figure 5c) and the decrease in cyclin B1 expression level (Figure 5d and Figure S1). Conversely, inhibition of CDK1 by RO-3306 caused mitotic slippage and accelerated the generation of these cells (Figure 5e). As expected, the combination treatment in the presence of paclitaxel instead of STLC also generated cells with aberrant-shaped nuclei (Figure S2). These results suggest that precocious degradation of cyclin B1 is a cause of mitotic slippage, generation of over-replicated cells, and the following cell death.

**Figure 6:**
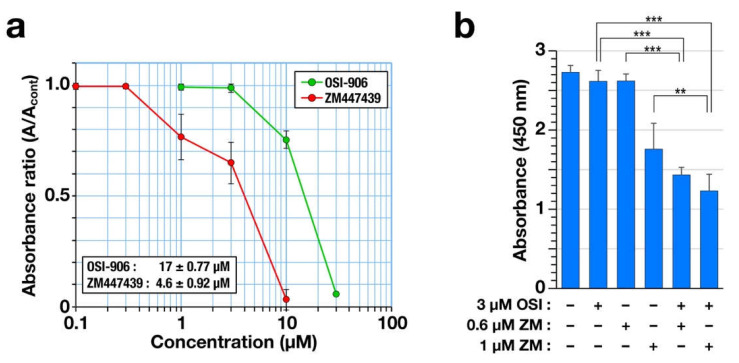
Combination of OSI-906 with ZM447439 reduces cell viability in the non-small cell lung cancer A549 cells. (**a**) The A549 cells were treated with OSI-906 or ZM447439 for 72 h, and cell viability was determined using a WST-8 assay. The relative absorbance ratios to the solvent control are plotted as mean ± SD calculated from three independent experiments. The IC_50_ was calculated in each experiment and is shown as mean ± SD. (**b**) The A549 cells were treated with OSI-906, ZM447439, or their combination for 72 h; next, a WST-8 assay was performed. The absorbance is plotted as mean ± SD calculated from more than three independent experiments. The *p*-values were calculated using Tukey’s multiple comparison test. ** *p* < 0.01; *** *p* < 0.001.

OSI-906 has been used in clinical trials for the treatment of lung cancer [21,22,23]. Therefore, the combination treatment effect on cell viability was examined for human non-small cell lung cancer A549 cells. First, a WST-8 assay was performed to determine the IC_50_ values. The absorbance of reduced 2-(2-methoxy-4-nitrophenyl)-3-(4-nitrophenyl)-5-(2,4-isulfophenyl)-2H-tetrazolium monosodium salt at 450 nm showed that both OSI-906 and ZM447439 reduced cell viability in a concentration-dependent manner. The IC_50_ values are shown in the graph (Figure 6a). Next, we examined the possibility of the OSI-906 and ZM447439 synergism using the methods reported by Chou and Talalay [24]. Although 3 µM OSI-906 showed almost no effect on cell viability, the combination with either 0.6 µM or 1 µM ZM447439 significantly reduced cell viability compared to the single treatment (Figure 6b). Combination index (CI) values were 0.32 (combined with 0.6 µM ZM447439) and 0.35 (combined with 1 µM ZM447439). The obtained values were less than 1, suggesting the possibility of OSI-906 and ZM447439 synergism for the reduction of cell viability in human non-small cell lung cancer A549 cells.

## 3. Discussion

In this study, we demonstrated that combination treatment of OSI-906 and ZM447439 causes cell death via generation of over-replicated cells with aberrant nuclei. Time-lapse imaging revealed that the defect in chromosome alignment and mitotic slippage are responsible for the generation of over-replicated cells. Treatment with the proteasome inhibitor and cyclin B1 staining revealed that precocious degradation of cyclin B1 is the mechanism underlying the generation of over-replicated cells. This study provides novel insights into combination chemotherapy using IGF1R inhibitor and Aurora B inhibitor.

The microtubule-targeting agents exert their anti-proliferative effects by activating the SAC via induction of incorrect bipolar kinetochore–microtubule attachments [25]. Unattached kinetochores trigger signals, including MPS1 kinase, for formation of the mitotic checkpoint complex (MCC) comprising Mad2, Bub3, BubR1, and CDC20 [19]. The MCC inhibits APC/C and thereby prevents the degradation of cyclin B1 and cohesin. This can halt cells before the onset of anaphase until all kinetochores are properly attached to microtubules emanating from two opposite poles. In our recent report, we demonstrated that IGF1R inhibition delays M phase progression via affecting the chromosome alignment. This delay is rescued by treatment of cells with the MPS1 inhibitor AZ3146, thus suggesting that activation of SAC is responsible for the delay in M phase progression and partly for the anti-proliferative effect of IGF1R inhibitor [16]. In contrast, the concentration of OSI-906 used for the combination treatment in this study is not too high to sufficiently delay chromosome alignment in RO-3306 synchronization experiment (Figure 3) and, as such, it caused a slight prolongation of M phase possibly via SAC activation (Figure 4). Given the delay in chromosome alignment in ZM447439-treated cells (Figure 3), 1 µM ZM447439 is likely to cause the activation of SAC rather than SAC inhibition. Therefore, it was possible to hypothesize that the anti-proliferative effects of the combination treatment would be attributed to the activation of SAC.

Gascoigne and Taylor proposed a model for deciding the fate of cells continuously treated with anti-mitotic drugs [26]. In this report, cell death pathway competes with the pathway for mitotic slippage. When degradation of cyclin B1 breaches the threshold for the mitotic exit earlier than breaching the threshold for cell death, cells prematurely exit the M phase, and the mitotic slippage prevents cell death in the M phase. When cyclin B1 levels are maintained above a threshold as a result of continuous SAC activation, mitotic slippage does not occur and the cell dies in the M phase. This means that the longer the cells are arrested in M phase, the more cell death occurs. Indeed, we recently reported that v-Src–induced mitotic slippage attenuates the cytotoxicity of paclitaxel [27]. ZM447439 prevents chromosome alignment [28]. Similarly, OSI-906 delays the onset of anaphase due to defect in chromosome alignment [16]. In the present study, a single treatment of either OSI-906 slightly or ZM447439 drastically prolonged the duration of metaphase (Figure 4). Furthermore, the combination severely disrupted the chromosome alignment (Figure 3). Therefore, we had expected that a combination would further delay the onset of anaphase and that cells would be arrested in M phase for longer periods with severe chromosome segregation defects. However, unexpectedly, generation of enlarged cells with aberrant nuclei (Figure 1) and over-replicated cells were observed (Figure 2), which indicates that the combination treatment caused SAC defect, which was supported by the result of the time-lapse imaging (Figure 4). Therefore, the anti-proliferative effects are not attributed to the prolonged duration of M phase due to SAC activation with the severe defect in the chromosome alignment.

How is mitotic slippage caused by the combination? Cyclin B1 staining showed that the combination treatment causes a decrease in cyclin B1 levels in the presence of the Eg5 inhibitor STLC. This can be caused by either inhibition of synthesis or acceleration of cyclin B1 degradation. Simultaneous treatment with the proteasome inhibitor MG132 partially reduced the number of cells with aberrant nuclei, thus suggesting that precocious degradation of cyclin B1 is responsible for the decrease in cyclin B1 levels and therefore the mitotic slippage. Considering that Aurora B plays a role in SAC activation via the recruitment of MPS1 [29,30,31,32,33], ZM447439 was expected to cause the precocious degradation of cyclin B1. However, M phase cells with low cyclin B1 levels were found in cells treated with OSI-906, but not with 1 µM ZM447439 (Figure 5). Furthermore, 1 µM of ZM447439 did not increase the number of cells after the onset of anaphase in the RO-3306 synchronization experiment (Figure 3). This implies that 1 µM ZM447439 is incapable of inhibiting the SAC function. The concentration of ZM447439 that affects chromosome alignment is expected to be lower than that inhibits the SAC function. ZM447439 at 1 µM may hinder the chromosome alignment without inhibiting SAC. These results raise the possibility that OSI-906 may partially disrupt the SAC function and synergize with ZM447439 to inhibit SAC function and accelerate the degradation of cyclin B1, thereby causing the mitotic slippage.

However, the MPS1 inhibitor AZ3146 does not enhance the anti-proliferative effect of OSI-906 [16]. Therefore, further defect in the SAC function was not the sole cause of the anti-proliferative effects of combination treatment. Mitotic slippage results in generation of over-replicated cells and the following cell division tends to be abnormal, thereby leading to aneuploidy [34]. Given that both OSI-906 and ZM447439 cause defects in chromosome alignment, the defects in chromosome alignment and the following mitotic slippage may generate cells with massive aneuploidy. When SAC is inhibited before chromosomes are properly attached to microtubules, chromosome bridges or lagging chromosomes can be trapped inside the intercellular canal. The presence of chromosomes inside the intercellular canal activates the abscission checkpoint, which prevents the progression of cytokinesis [35,36]. Aurora B is reportedly involved in this checkpoint [35]. Thus, upon Aurora B inhibition, even if chromosomes would exist inside the intercellular canal, the abscission checkpoint is not activated and is not capable of preventing progression of cytokinesis, thus resulting in cleavage furrow regression. Alternatively, even if chromosomes are segregated in anaphase, inhibition of Aurora B leads to cytokinesis failure, since Aurora B plays a role in cytokinesis [37,38]. This cytokinesis failure generates cell with massive aneuploidization and chromosomal instability (CIN). Although CIN at a minor levels is thought to drive tumorigenesis, an increase in CIN may suppress cancer cell proliferation [39,40]. Therefore, SAC defect following severe chromosome alignment defect may generate massive aneuploidy in combination-treated cells. Induction of CIN at higher levels may be responsible for the increase in cell death caused by the combination treatment of Aurora B and IGF1R inhibitors, which is supported by the report describing that tetraploidization increases sensitivity to Aurora B inhibitors [41].

In certain cancers, IGF1R signaling contributes to oncogene-induced cancer transformation, thus suggesting that IGF1R signaling pathway is an attractive therapeutic target for cancer chemotherapy [42]. Because of expression of IGF1R in many human tissues and cell types, as well as cross-reaction of many IGF1R inhibitors with insulin receptor, dose reduction of IGF1R inhibitors is required to reduce the side effects. In this regard, combination therapy is advantageous for dose reduction and combination therapy approaches have been widely applied [43]. However, some phase II trials of linsitinib (OSI-906) alone or in combination with other anticancer drugs have failed to demonstrate clinical activity as cancer chemotherapy [21,22,23,44,45,46]. Many Aurora kinase inhibitors have been also developed, but not yet approved for clinical use due to their cytotoxicity. In this study, combination of IGF1R inhibitor and Aurora B inhibitor drastically reduced cell viability and induced cell death. Conversely, each inhibitor alone did not reduce cell viability at the concentration used in this study. Therefore, this combination can potentially reduce their concentrations and side effects. In addition, the reduction of the doses of these agents in cancer chemotherapy will enable further combinations with other anti-cancer agents, such as anti-microtubule agents.

Microtubule polymerization and depolymerization are critical for mitotic spindle formation. The anti-microtubule agent paclitaxel suppresses microtubule dynamics, activating SAC via defect in mitotic spindle formation. While SAC-caused prolonged mitotic arrest is required for paclitaxel-induced cell death, SAC signaling is partially disrupted in many cancer cells [47]. Thus, an anti-microtubule-based chemotherapy is not efficient because of failure in prolonged mitotic arrest. It is noted that a severely disabled SAC signaling causes cell death due to massive aneuploidy [47]. In this study, the combination treatment induced mitotic slippage even in the presence of paclitaxel, as shown in Figure S2. Treating cancer patients with a combination of OSI-906 and ZM447439, together with anti-microtubule agents, can cause mitotic slippage and generate cells with aberrant-shaped nuclei with over-replicated DNA, inducing cell death possibly via massive aneuploidy. Therefore, in addition to anti-microtubule-based chemotherapy, treatment with a combination of OSI-906 and ZM447439 can be a novel and fruitful approach for cancer chemotherapy. Further study is required to determine whether a combination of OSI-906 and ZM447439 actually accelerates anti-microtubule agent-caused cell deaths.

## 4. Materials and Methods

### 4.1. Cells

Human cervix adenocarcinoma HeLa S3 cells and human non-small cell lung cancer A549 cells (Japanese Collection of Research Bioresources, Osaka, Japan) were maintained in Dulbecco’s modified Eagle’s medium supplemented with 20 mM 4-(2-hydroxyethyl)-1-piperazine ethanesulfonic acid (HEPES)-NaOH (pH 7.4) and 5% FBS. No Hoechst 33342 fluorescence was observed in the cytoplasm, indicating that these cells were not infected with mycoplasma. 

### 4.2. Chemicals

The IGF-1R inhibitor OSI-906 (07333, LKT Laboratories, St. Paul, MN, USA), the Aurora B inhibitor ZM447439 (JS Research Chemicals Trading, Wedel, Germany), the proteasome inhibitor MG132 (3175-v, Peptide Institute, Osaka, Japan), the Eg5 inhibitor STLC (164739, MilliporeSigma, Burlington, MA, USA), and the microtubule-targeting agent paclitaxel (169-18611, FujiFilm Wako, Osaka, Japan) were used in this study. The CDK1 inhibitor RO-3306 (S7747, Selleck Chemicals) was used for synchronization to M phase [48]. Dimethyl sulfoxide (Nacalai Tesque, Kyoto, Japan) was used to dissolve these chemicals.

### 4.3. Antibodies

The following primary antibodies were used for immunofluorescence (IF) and immunoblotting (IB): mouse monoclonal anti-γ-tubulin antibody (IF, 1:250; GTU-88, Sigma-Aldrich, St. Louis, MO, USA), rat monoclonal anti-α-tubulin antibody (IF, 1:800; IB, 1:4000; YOL1/34, MCA78G, Bio-rad, Hercules, CA, USA), rabbit polyclonal anti-cyclin B1 (IF, 1:250; IB, 1:2000; H-433, sc-752, Santa Cruz Biotechnology), and anti-cleaved caspase-3 (IF, 1:500; #9661, Cell Signaling Technology, Danvers, MA, USA) antibodies. 

The following secondary antibodies for IF staining were purchased from Thermo Fisher Scientific (Waltham, MA, USA): Alexa Fluor 488-labeled donkey anti-mouse immunoglobin G (IgG) (1:800; A21202), Alexa Fluor 488–labeled donkey anti-rabbit IgG (1:800; A21206), Alexa Fluor 488–labeled donkey anti-rat IgG (1:800; A21208), and Alexa Fluor 555–labeled donkey anti-rabbit IgG (1:800; A31572) antibodies. The following secondary antibodies for IB were purchased from Jackson ImmunoResearch (West Grove, PA, USA): horseradish peroxidase-conjugated anti-rabbit (1:8000; 711-035-152) and anti-rat (1:8000; 712-035-153) IgG antibodies.

### 4.4. Immunofluorescence Microscopy

Cells were stained according to previously described methods [49,50,51]. In brief, after washing with phosphate-buffered saline (PBS) (–), cells were fixed with 4% formaldehyde in PBS (–) for 20 min, permeabilized, and blocked with PBS (−) containing 3% BSA and 0.1% saponin for 30 min at room temperature. When M phase cells were observed, the washing step was omitted. For staining of γ-tubulin, cells were fixed with methanol for 5 min at −30 °C. For staining of cleaved caspase-3, loosely attached or floating cells were sedimented onto glass cover slips by centrifugation prior to fixation. Primary and secondary antibodies were diluted with PBS (–) containing 3% BSA and 0.1% saponin and applied for 1 h each at room temperature. Hoechst 33342 was used to stain DNA with the secondary antibody. Fluorescence images were obtained with a fluorescence microscope (IX-83, Olympus, Tokyo, Japan) fitted with 20 × 0.45 NA, 40 × 0.75 NA objective lenses, and 60 × 1.42 NA oil-immersion objective lens (Olympus). Hoechst 33342, Alexa Fluor 488, and Alexa Fluor 555 signals were captured using a U-FUNA cube (360–370 nm excitation, 420–460 nm emission), U-FBNA cube (470–495 nm excitation, 510–550 nm emission), and U-FRFP cube (535–555 nm excitation, 570–625 nm emission), respectively. Images were processed using ImageJ (National Institutes of Health, Bethesda, MD, USA), Photoshop CC, and Illustrator CC software (Adobe, San Jose, CA, USA).

### 4.5. Western Blotting

Cells were lysed in sodium dodecyl sulfate (SDS)–sample buffer containing protease and phosphatase inhibitors (2 µg/mL aprotinin, 0.8 µg/mL pepstatin A, 2 µg/mL leupeptin, 2 mM PMSF, 20 mM β-glycerophosphate, 50 mM NaF, and 10 mM Na_3_VO_4_). The cell lysates were subjected to SDS–polyacrylamide gel electrophoresis and were electrotransferred onto polyvinylidene difluoride membranes (PVDF; Pall Corporation, Port Washington, NY, USA). After blocking with Blocking One (Nacalai Tesque) at room temperature for 30 min, the membranes were incubated with the antibodies diluted with Tris-buffered saline [20 mM Tris–HCl (pH 7.5), 137 mM NaCl] containing 0.1% Tween 20 and 5% Blocking One. Chemiluminescence was detected with ChemiDoc XRSplus image analyzer (Bio-Rad) using Clarity (Bio-Rad) as the chemiluminescence substrate.

### 4.6. Cell Cycle Synchronization

To analyze the progression of M phase, cells were treated with 6 µM RO-3306, a CDK1 inhibitor, for 20 h and then washed with pre-warmed PBS containing Ca^2+^ and Mg^2+^ [PBS(+)] four times on a water bath at 37 °C. Immediately, cells were incubated with pre-warmed medium for 60 min in a CO_2_ incubator. After fixing with 4% formaldehyde in PBS (–), cells were stained for α-tubulin and DNA. Based on the morphologies of mitotic spindle and chromosomes, cells were classified into four groups: prophase/prometaphase (P/PM), metaphase (M), anaphase/telophase (A/T), and cytokinesis (Cyto).

### 4.7. Time-Lapse Imaging

For time-lapse imaging of M phase cells [52], cells were synchronized to M phase as described above. After removal of RO-3306 by washing the cells, 0.1 µM Hoechst 33342 was added into the culture and time-lapse images of bright-field and Hoechst 33342 fluorescence were acquired in a live-cell chamber at 37 °C in 5% CO_2_ using the Operetta imaging system (PerkinElmer, Waltham, MA, USA). The durations of each of the mitotic phases, such as P/PM, M, and A/T/Cyto, were determined.

### 4.8. WST-8 Assay

Cell viability was assessed using a Cell Counting Kit-8 (Dojindo, Kumamoto, Japan) based on the manufacturer’s instructions, as described previously [14]. A549 cells seeded in 96-well plates (3000–4000 cells per well) were cultured with either OSI-906, ZM447439, or their combination for 3 days. DMSO was used as a solvent control. The absorbance (450 nm) of reduced 2-(2-methoxy-4-nitrophenyl)-3-(4-nitrophenyl)-5-(2,4-isulfophenyl)-2H-tetrazolium, monosodium salt (WST-8) was measured using an iMark Microplate Reader (Bio-Rad).

### 4.9. Combination Index

Combination index (CI) was calculated by the following equation [24]:

CI = (D)_1_/(D_x_)_1_ + (D)_2_/(D_x_)_2_

where (D_x_)_1_ and (D_x_)_2_ are the concentrations for OSI-906 and ZM447439 alone, respectively, that cause x% suppression of cell viability. (D)_1_ and (D)_2_ are the concentrations of OSI-906 and ZM447439, respectively, in combination that also cause x% suppression. (D_x_)_1_ and (D_x_)_2_ were predicted from the results of dose-response curve shown in Figure 6a. CI values less than 1 suggest synergism.

### 4.10. Statistics

Bartlett’s test was used to examine the homogeneity of variance. One-way analysis of variance was used to identify the statistical significance for multiple comparisons with equal variances, followed by Tukey–Kramer test. For multiple comparisons with unequal variances, the Games–Howell test was used. Analyses were performed with EZR (Saitama Medical Centre, Jichi Medical University; http://www.jichi.ac.jp/saitama-sct/SaitamaHP.files/statmedEN.html [53] accessed on 1 May 2021), which is a graphical user interface for R (The R Foundation for Statistical Computing, Vienna, Austria, version 2.13.0).

## Supplementary Materials

The following are available online at https://www.mdpi.com/article/10.3390/ijms22115706/s1.
