# Supplementary material for: Combination Treatment of OSI-906 with Aurora B Inhibitor Reduces Cell Viability via Cyclin B1 Degradation-Induced Mitotic Slippage
Source: Int J Mol Sci. 2021 May 27;22(11):5706. doi: 10.3390/ijms22115706 (PMC8197973; doi:10.3390/ijms22115706)
Supplement: Supplementary file 1 [file ijms-22-05706-s001.zip › ijms-1184041-supplementary.pdf]

a

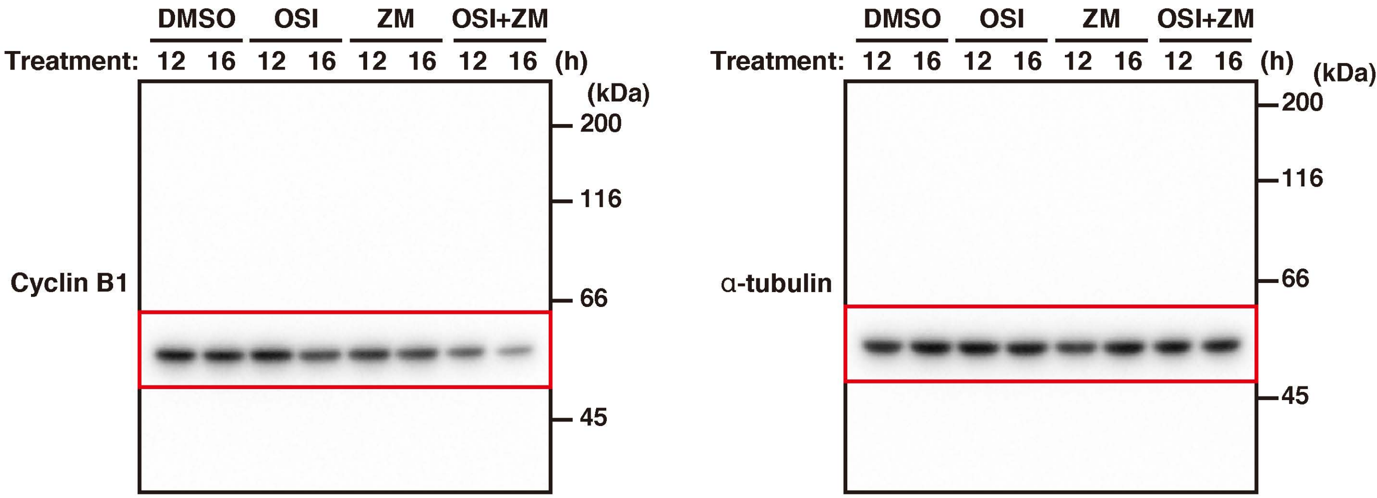

Full-length blots of Figure 5**b**.

b

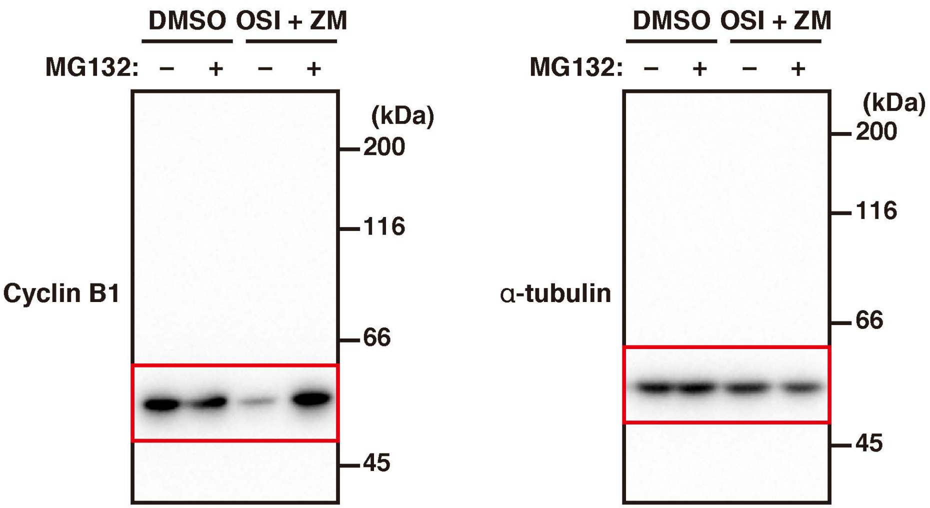

Full-length blots of Figure 5**d**.

Figure S1. Full-length blots of Figure 5b and d.

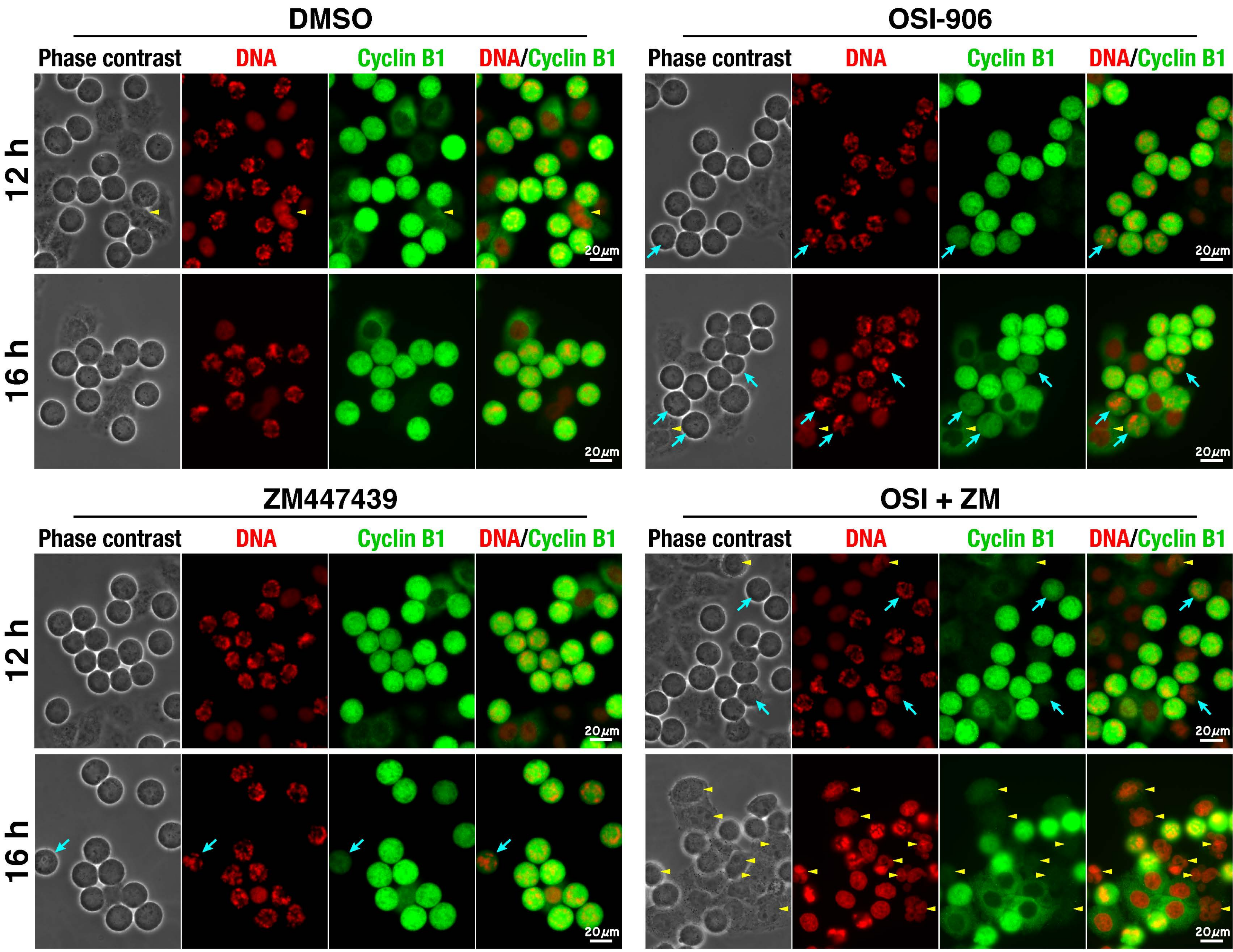

Figure S2. Combination of OSI-906 and ZM447439 in the presence of paclitaxel generates cells with aberrant-shaped nuclei via mitotic slippage. HeLa S3 cells were treated with 0.5 μg/ml paclitaxel in the presence of OSI-906, ZM447439, or their combination (OSI + ZM). DMSO was used as solvent control. At 12 and 16 h after the start of treatment, cells were fixed and stained for cyclin B1 (green) and DNA (red). Represent images are shown. Blue arrows indicate M phase cells with lower expression levels of cyclin B1. Yellow arrowheads indicate cells wit aberrant nuclear morphologies. Scale bars, 20 μm.
